# Social Determination of HIV: Women’s Relationship Work in the Context of Mass Incarceration and Housing Vulnerability

**DOI:** 10.1007/s10461-021-03238-4

**Published:** 2021-04-01

**Authors:** Kim M. Blankenship, Alana Rosenberg, Danya E. Keene, Akiv J. Dawson, Allison K. Groves, Penelope Schlesinger

**Affiliations:** 1 Department of Sociology, American University, 4400 Massachusetts Ave NW, Washington, DC 20016-4072, USA; 2 Department of Epidemiology of Microbial Diseases, Yale School of Public Health, Yale University, New Haven, CT, USA; 3 Department of Social and Behavioral Sciences, Yale School of Public Health, Yale University, New Haven, CT, USA; 4 Department of Sociology, Howard University, Washington, DC, USA; 5 Department of Criminal Justice and Criminology, Georgia Southern University, Statesboro, GA, USA; 6 Department of Community Health and Prevention, Dornsife School of Public Health, Drexel University, Philadelphia, PA, USA

**Keywords:** Social determinants, Social determination, Mass incarceration, Housing, Gender, HIV

## Abstract

We contrast a typical “social determinants of health” framing with a more dynamic and complex “social determination of health” framing to analyze HIV-related sexual risk among women in low-income, segregated neighborhoods in New Haven, CT. Using an abductive approach, we analyze repeated, longitudinal qualitative interviews conducted over a 2-year period with a sample of 14 HIV-negative women who engaged in sex with men during the study period. Three case studies are presented to demonstrate how behaviors and sexual practices typically described as HIV “risks” can be understood as part of the work of establishing and maintaining monogamous committed relationships, which we call “relationship work,” shaped in a context characterized by housing vulnerabilities and the many manifestations of mass incarceration and the surveillance state. We conclude by suggesting that for these women, their relationship work is the work of HIV prevention and life in low-income segregated neighborhoods is their HIV-related risk.

## Introduction

A “social determinants of health (SDOH)” framing focuses attention on how health is shaped by social factors [1–3]—for example, access to economic resources, neighborhood and housing conditions, quality of health services—and often underlies the call for structural interventions to address health [4]. As such, it emphasizes that behavioral and biomedical determinants are located in broader social structures. Still, its operationalization often proceeds without explicitly theorizing about the “social” or how it operates to determine health [5, 6]. Analyses frequently adopt an ecological framing whereby “risk environments” are characterized by various factors considered “social” largely because they are “outside the individual” or “upstream” from individual behaviors. These “social” factors are analyzed as independent of each other and operating along linear causal paths to impact health outcomes, often through their impacts on individual behaviors (for examples in HIV [7–10]). The health behaviors of interest are also viewed as independent of and predicted by these social factors, rather than as embodying social meanings and practices.

Critiques of this ecological framing focus on its conceptualization of determinants and of causality. Krieger [11], for example, calls for abandoning the “upstream”/“downstream” distinction among social determinants because it obscures the fundamental connectedness among critical determinants and between determinants and outcomes, and thereby hides how their interconnections represent processes of politics, power and domination. Less well known but also more theoretically explicit is the call of the Latin American Social Medicine and Collective Health for a “social determination of health” framing, which views the process of social determination as a dynamic one fundamentally shaped by structures of power and oppression operating along race/ethnic, class, and gender lines [12, 13].

We analyze HIV-related sexual risk within a dynamic social determination framing. We focus on the intersection of housing vulnerabilities and mass incarceration, “social determinants” we intentionally selected as contemporary manifestations of deeply rooted structures that both reflect past and create and perpetuate new forms of race, class, and gender inequality in the United States. With regard to housing, a growing literature [14–16] situates such things as residential displacement and segregation, and race inequities in wealth accumulation, in a long history of racist housing policies and practices enacted through racist politics. Reid [17] adds to this understanding of the history of housing vulnerabilities by tracing its roots in gender and class, as well as race interests.

With regard to mass incarceration, both Alexander [18] and Wacquant [19] have argued that mass incarceration [or hyper-incarceration (Wacquant)]—signified most immediately by both rates of incarceration in the US that are higher than those in any other country and substantial race inequities in these rates [20]—represents a contemporary structure of racial domination that emerged from a long history of racist practices, policies, and politics tracing back to slavery. And sociologist George Lipsitz [21] brings housing and mass incarceration together, arguing that a full appreciation of mass incarceration and its impacts must simultaneously attend to housing insecurity and economic inequality. He traces in their historical intersection, the multiple forms of raced and gendered exploitation they represent as they are, in turn, “inscribed inside the routine practices of contemporary capitalism” (p. 1747).

Housing vulnerability and mass incarceration have each been linked to various health outcomes (e.g. [22–24]). Most research on HIV and housing focuses on housing as a social determinant of risk behaviors, treatment adherence, or health outcomes among people living with HIV. In a recent review, Aidala et al. [25] confirm the substantial impact of housing on these outcomes and suggest that changes to housing environments represent “possible and promising” HIV-related interventions (p. e19). A smaller literature explores the association between housing and HIV-related risk among uninfected vulnerable populations. It shows, for example, that eviction [26] and forced moves [27] are associated with HIV/STI-related prevalence and risk, respectively. It also has analyzed risk behaviors, prevention knowledge, and HIV diagnoses among the homeless [28–30].

There is growing attention to the relationship between mass incarceration and HIV/AIDS, most of which has operationalized mass incarceration as incarceration rates or focused on the impacts of self or partner incarceration on HIV risk. Early work documented population level associations between incarceration and HIV prevalence [31–33]. More recently, studies have sought to identify the pathways through which incarceration may lead to HIV. Many of these have analyzed self or partner incarceration and have focused on the impacts of incarceration on partner turnover, and relationship instability, concurrency, and/or dissolution [34–38]. Qualitative data contributes further to understanding these impacts. For example, Cooper et al. [39] adopt a social ecological framework in analyzing longitudinal qualitative interviews with African American women whose partners were recently incarcerated. Accordingly, they examine “structural-, community-, relationship-, and personal-level” factors that impact sexual risk trajectories among women following a partner’s incarceration.

Widman et al. [40] have connected these literatures by analyzing the intersecting impacts of incarceration and housing instability on sexual risk behaviors. Using cross-sectional data they find that, among African American STD clinic patients, those with a history of both incarceration and housing instability had more sexual partners and more unprotected sex than those without such histories. Still, much more research is needed to illuminate the linkages among mass incarceration, housing, sexual practices, and HIV risk.

In the following analysis we expand this understanding by exploring how deeply rooted structures of inequality, currently represented by mass incarceration and housing vulnerabilities, intersect to impact the relationships, and sexual practices within them, of women living in low income racially segregated neighborhoods. We do so while simultaneously advancing a social determination framing for understanding these impacts. Accordingly, our focus is not on distinguishing factors at different levels of analysis and identifying distinct pathways through which a particular instance of incarceration (of self or partner) may combine with these factors to affect risk outcomes. Instead, we develop a more wholistic and dynamic understanding of how women experience life in communities impacted by mass incarceration and housing vulnerability—where residents move in and out of the criminal legal system and, correspondingly, leave and then return to their communities, often subject to the demands of probation or parole; where their streets, schools, and homes are regularly surveilled; where safe, affordable housing is scarce and having a criminal record can determine access to housing, as well as jobs and other conditions for livelihood; and where their own and the incarceration of their family and loved ones may further complicate their lives.

Through analysis of longitudinal qualitative interviews conducted with these women over 2 years, we also expand this literature by redirecting attention from the typical focus on risks associated with no or inconsistent condom use and relationship instability, dissolution, and concurrency. Our analysis reveals instead, both the value our respondents place on long term monogamous committed relationships (the safest types of relationships from the standpoint of HIV) and the extraordinary efforts they make to find and maintain such relationships. We call this “relationship work.” And, we suggest that sexual practices (typically labelled as “risk behaviors”) that occur in these relationships must be understood with attention to the meaning they hold for and are given by this relationship work and as embedded within contexts shaped by mass incarceration and housing vulnerabilities.

## Methods

We draw on data from the NIH-funded Justice, Housing and Health Study (JustHouHS), whose primary purpose is to explore the intersecting impacts of housing and mass incarceration on sexual health and HIV-related sexual practices. We enrolled 400 low-income residents of New Haven, CT, half of whom were released from prison within the past year. Participants took a baseline survey in fall to winter of 2017–2018 and returned for four six-month follow-up surveys. A subset of purposively sampled participants (n = 54) completed five qualitative interviews every 6 months. Three co-authors (Rosenberg, Schlesinger, and Keene) conducted these interviews. The study was approved by the Yale University Institutional Review Board (IRB), which also served as the designated IRB for American University and Drexel University, and received a Certificate of Confidentiality.

Semi-structured interviews averaging an hour in length included topics related to current and prior housing, criminal legal (CL) involvement of self, partners, and family, experiences with and attitudes about policing, economic situation, interactions with social services, sexual relationships, condom use, and HIV testing. Follow-up interviews elaborated on previous interviews, described changing circumstances, and highlighted new topics. All interviews were coded using broad index topics (e.g. housing, CL experience, partners). We created a matrix for each participant that summarized each topical area per interview wave, and a master matrix that compiled summaries for topics across participants for all waves (see [41] for more detailed discussion).

The qualitative sample included 17 women. For the present analysis, we focused on those who self-reported being HIV-negative and having sex with men during the study period (N = 14). For each respondent, the first and fourth authors read each interview and wrote and discussed extensive analytical memos focused on the themes of housing, CL experience and relationships. The first author then reviewed data coded at the intersection of housing, relationships, and HIV for each of the 14 women and wrote additional analytical memos, working iteratively between the coded data, transcripts, and matrices. She then discussed these memos with co-authors to clarify uncertainties regarding specific details of events or descriptions and to address differences in interpretation and meaning.

Through this intensive, abductive process [42] a clear theme emerged from the women’s narratives: each indicated that they placed a high value on and had a strong desire for a long-term committed partnership. Furthermore, at some point during the study period they described being in and working to maintain, and/or working to establish, and/or working to successfully end such a relationship. We came to understand this as “relationship work”. To best present the complex ways that their relationship work was shaped by mass incarceration and housing vulnerabilities, and their sexual practices were given meaning within their relationships, we selected three cases to represent distinct efforts to develop, maintain, and/or successfully end relationships. The first author then created additional longitudinally organized analytical memos for these cases using the same iterative process described above. She discussed drafts with the second and last authors who had conducted the interviews with these women. Any differences of interpretation were discussed and resolved.

In what follows, we describe the relationship work of three women. In keeping with the case study approach, we focus on providing an analysis that is valid and consistent with our framing, rather than on achieving “representativeness” [43]. The women’s names are pseudonyms chosen by them. Two of them only referred to their partners as “husbands”; that is how they are represented in the analysis. When women referred to partners by name, we designated pseudonyms for them. Ages are at baseline.

## Case Studies

### Lily

Where he going? He ain’t going nowhere all this time

Lily is a 43 year-old African American woman with 5 children. Her oldest two boys share a father with whom she has had little contact since they became adults. The father of her other three children, a teenage boy and two younger girls, is her husband; they’ve been together for more than 15 years and married for nine. Prior to the study, Lily and her husband had recently experienced a serious disruption in their relationship leading to separate households in different neighborhoods; Lily in one apartment and her husband and their three children in another. According to Lily, the separation of households occurred because, during an argument, “he said the wrong thing and I gave him a nice whack.” He called the police, resulting in her incarceration for 3 weeks on a domestic violence charge before being released on 18 months of probation, a violation of which would send her back to prison.

It is clear across the interviews that though angry at her husband for calling the police, which she saw as a betrayal, Lily worked to maintain their committed relationship, simultaneously ensuring her children’s welfare. “Yeah [I was mad], but…that’s why I moved….’Go get your apartment, take the kids, and I’m going to a one-bedroom… I made sure my kids—I handled them straight.” She explains that her husband: “used to be like…’You ain’t have to put us out—’I said I didn’t put my kids out. … ‘You had to go because you had me locked up.’” Though sending her children to live with her husband was difficult for Lily, it ensured they were cared for both while she was in prison, and after her release while she and her husband worked things out.

Lily’s efforts to find a job over the study period were unsuccessful, as she had no recent formal work history (previously taking care of the family while her husband worked) and now, had a criminal record. Despite many applications, she never made it past the background checks. Her precarious financial situation meant that Lily could not support her children economically. However, during the separation she continued to care for them; they stayed with her over weekends and most of the summer. Lily explained: “Well it [the separation] ain’t really no break…they my kids, you know, so I don’t expect to not still be mommy.” Throughout the study period, Lily’s search for a place where they could all live together was also driven by her attention to the children’s well-being. Her housing voucher had been re-certified, enabling her to receive a housing subsidy for another 3-bedroom place in the private housing market. But her efforts to find somewhere meeting all of her priorities—in a safe neighborhood, where the children could stay in their same schools, with the same landlord, and equipped with critical amenities—remained unsuccessful.

Though maintaining their relationship, the time Lily and her husband actually spent together was limited while she was on probation. At one interview she explains why she rarely went to his place: “I was on … probation, and remember he was the victim so…if he trigger me off and I do something he could call the cops and who going to jail? Me. So….that was a smart thing to do, stay away…but now since I’ve been off probation… if we get into a argument I don’t have to hear about, ‘oh, you’re the one that’s on probation.’” Indeed, the first Friday after her probation ended, they went to breakfast together. But before it ended, the limited contact with her husband protected her from returning to prison for 18 months: “I wouldn’t want to leave my kids like that [for 18 months….]”.

At baseline, Lily indicates that in spite of the separation of households, she and her husband are in a committed relationship: the separation is meant to preserve that relationship. When asked, “you think you’ll stay together?” she replies: “Where he going? He ain’t going nowhere all this time and I ain’t going nowhere all this time ….” Still, during the initial separation period their limited time together meant they had sex infrequently. She believes he’s “OK with that. ‘Cause he be at work…He’s busy…” Protecting herself from the possibility of reincarceration by spending long periods of time away from her husband and caring for the children, Lily gives him free time he might not otherwise have. This does not mean he is involved with another woman; Lily believes he is “too busy” for that. But it does raise that possibility. Yet, when they resume more regular sexual activity after her probation ends, Lily indicates that they do not use condoms: “I tell him to put a condom on, he’ll really bug out… he’ll look at me like—that’d be a argument…Or be thinking I’m sleeping with somebody else. You know how men think. And I would think that too if he was to [say], ‘I’m gonna put on a condom.’”.

Though they face challenges as they deal with the consequences of her incarceration, and as she protects herself from further involvement with the CL system and seeks suitable and affordable housing for her family, Lily and her husband manage to maintain their committed relationship and protect their children’s well-being. This was likely possible, in part, because they each had independent access to housing, she through a voucher she has had since she was 19 and he, with no recent CL history, through earnings from his job. They have also managed to financially support two separate households, although by her last interview, her own financial situation had become more precarious; Lily was still without work, but incurring costs supporting her oldest son who was recently arrested.

### Maya

But I’m his wife…It’s like a bond … even his family won’t take care of him like I will

Maya is a 44-year-old woman who identifies as racially mixed. At baseline she is about 7 years into a relationship with the man she refers to as her second husband. She has four adult children and one teenager, but mentions having little contact with them during the study. Maya’s husband, who is in recovery from addiction, is disabled and requires hemodialysis 3 days per week. Through much of the study period, Maya works to preserve her commitment to her husband, which includes a strong sense of responsibility for his health and well-being.

Not long before she entered the study, Maya had been arrested on drug sales charges. She reported that she received excessive attention for selling some drugs from her “private stash” to a friend. “I mean they treated me like I was the queen-pin of [the town]… the whole police force came to take me out of my house. They made a big to-do out of it and I’m like, ‘I’m a drug addict. I was just trying to make money to pay a bill.’” However minor Maya believed her infraction to be, the consequences of her incarceration for her well-being and her relationship were substantial; she struggled throughout the study with recovery from addiction, community supervision, housing and employment challenges.

After 2 months in prison awaiting sentencing and 2 months in a transitional treatment program, Maya accepted a judge’s offer of release onto probation. She told the judge her husband needed her to take his medicine properly and to go to dialysis regularly. She was also “scared he might slip back into his old self and start using drugs… I’m his wife. You know?.. [W]hen you get married, it’s just you and your husband…It’s like a bond … even his family won’t take care of him like I will.”

Maya’s husband brought $850 in monthly disability benefits to their relationship. Additionally, his disability had qualified him for an apartment in a Housing Authority (HA) building in a suburb where they had been living when she was arrested. After her incarceration, she returned to live with him there, but the HA insisted her husband remove her from the lease. He refused and they were evicted; his eligibility for housing was not transferable. For nearly a year they moved around, essentially homeless, she staying in sober houses and other programs or joining him on the couches of relatives and friends. Maya describes the frustrations of trying to coordinate a job search, a search for housing, the demands of probation, and the responsibilities of caring for her husband. In 3 months, she submitted 73 job applications, getting 12 first and no second interviews, largely due to failing background checks. She eventually found a job at a cleaning company through her church networks. This meant she had more resources to put towards housing. But it made her housing search challenging in other ways: “I have to take off work to find a house, but then I need to work to pay the rent.”

Nine months after her baseline interview, Maya again worked her church networks and found an apartment with a (high) rent that reflected the landlord’s willingness to “ignore” their eviction history. It also required a substantial security deposit. And, she described extensive drug selling in the surrounding neighborhood—hardly ideal for a couple trying not to use drugs. Still, she remained hopeful that they could make it work because her husband had his disability checks and though unfair work practices led her to quit her job at the cleaning company, she maintained some cleaning jobs “on the side” and continued to search for work.

This strategy, however, was interrupted when her husband went to the ER and ended up having heart surgery. Her caretaking and commitment to the relationship meant that she missed a promising second job interview and could not continue either her job search or cleaning work. Unable to keep up with the rent, the landlord agreed they could break the lease. Maya went to live with her aunt and he with his cousin. She insisted that her plan was to return and stay with him: “I’ll never leave him … I love him, but I needed this break.” And yet, she also found herself wondering whether they could make it work: “He’s like, ‘Just come stay here [with him at his cousin’s] and in 3 months we’ll save some money and… we’ll be able to get a place.’ But see, he’s not looking at the whole thing. Like, ‘okay, we saved money last time. Look how hard it was for us to even get somebody to show us an apartment.’”

Maya does leave her husband. It starts when she encounters an old boyfriend that she left 18 years ago, but whom she refers to as “the one that got away.” His sister tells Maya, “Do you know he’s still in love—he’s been in love with you for all this time.” At her third follow up interview, Maya discusses the overlapping dissolution of her relationship with her husband (referred to as “ex-husband” in the fourth interview) and the development of her relationship with her boyfriend Curtis (referred to as “husband” in the fourth interview). Though initially presenting the start of the new relationship as an impulsive move to reconnect with a past love, in a later interview she names her ex-husband’s resumption of drug use as a final factor in the relationship’s dissolution. She recounts his many health issues and then: “Either way, he relapsed and I just can’t—I will not sit there and watch you kill yourself. I love him to death but I can’t do it….’Cause if something happens I wouldn’t be able to live with myself.” Her husband’s resumption of drug use suggested to Maya that she had failed in her commitment to him, but she also recognized that staying with him jeopardized her own recovery success. Curtis, with already proven feelings for her, signified a chance to try again in a new relationship.

Despite the many challenges to her work maintaining their relationship, until Maya left her husband, she was deeply committed to it. They remained sexually active with one another during their time together: “Me and him [her husband], we never use condoms and we don’t because we’re monogamous.” Yet, as part of her commitment to him she explains: “I don’t hide anything from my husband,” including her initial “hook up” with Curtis. In the same conversation declaring their monogamy, she also indicates that she suspects her husband was having sex with other women during the periods when they were living separately: “He’s a man. You know? Men are simple…It’s not rocket science. What you won’t do, another woman will.” Nevertheless, Maya describes their agreed upon “rules”: “Do what you gotta do. Make sure you put on a condom and you know the rest of the rules. … No penetration [without a condom]. Oral sex is all right.” Because sex between her and her husband is embedded within a committed relationship that includes open communication and clear behavioral expectations, condoms are perceived as unnecessary. Sex with other partners, when following their agreed-upon rules, does not threaten this monogamous relationship.

Not long after her first “hook up” with Curtis, their relationship began progressing towards one of commitment, developing with a similar emphasis on caretaking; indeed, Curtis had serious COPD (chronic obstructive pulmonary disease). With regard to sex, while Maya followed the rules she had established with her husband by using condoms the first time she had sex with Curtis, as the relationship with him quickly progressed, they abandoned them. Still, her last interview made clear that the work to develop their committed relationship was still ongoing. Less than a year after they got together, Maya, interpreting Curtis’ lack of interest in sex as infidelity (she later learned it was because of his worsening COPD) became distressed, resumed using drugs, and failed her drug test. To avoid a possible 7 years in prison, she agreed to enter an in-patient program even while acknowledging that doing so might jeopardize the progression of her relationship with Curtis.

### Rayna

You were supposed to jump along with me…to be by my side, not in front of me, not behind me

Rayna is a 43-year-old African American woman. She has five children, an adult daughter and mother of her 1-year-old grandson, and four sons (three teenagers and an 8-year-old). Essentially, Rayna has been singularly responsible for these children. Their four different fathers have been in and out of the CL system and have had little involvement in their lives. Her longest relationship (10 years, albeit interrupted by his incarceration) was with the abusive father of her two middle sons. Rayna has never been incarcerated.

Over the study period, Rayna juggles overlapping relationships as she works to determine if either of them has the potential to meet her ideal of a “forever partner”: an intellectual companion who provides interesting conversation but also shares the pleasures of simple activities like bike rides and breakfasts together. “Trustworthiness,” including sexual monogamy, is also fundamental to this ideal. Rayna’s relationship work also involves ensuring her highest priority: her children’s well-being. To this end, she works to preserve access to safe and affordable housing while successfully navigating the CL and social service systems. She fiercely protects hers and their well-being, taking swift action in the face of threats, sometimes in ways that impede her relationships.

Rayna has had a Section 8 housing voucher since she was 17. While giving her access to affordable housing, it has not always ensured her family’s safety. At baseline, she is living with her boys in a relatively quiet New Haven neighborhood in a 4-bedroom unit in a 3-family house. The rocky road that brought her here included, in 2015, spending the holidays in a 90-day shelter, where she and her children were temporarily relocated by Section 8′s domestic violence services to protect them from retaliation when she rejected an offer to join a gang. On the next Thanksgiving, four weeks after moving into a different apartment, she describes how her turkey basting is interrupted by a murder outside her kitchen door. “I hear’No, no, pop, pop, pop, pop.’ I was like, oh my God, and then I looked in my son’s room, ‘cause I thought maybe somebody shot in his window ‘cause they were right there….” This time, Section 8 is less helpful. They review the record of domestic disturbances and complaints associated with several previous abusive partners and suggest she may be the “troublemaker,” placing her in jeopardy of losing her voucher altogether. When she involves Legal Aid, they allow her to keep the voucher but not to break the lease to relocate.

At baseline, Rayna’s primary complaint about her housing is that it’s too small. She longs for a place that will facilitate family activities, and in one interview she excitedly anticipates life in a new apartment complex where she is about to move. On the top floor, she describes, there is a common area where she and the boys can gather for family nights of games and prayer. But it turns out to be much less than she had hoped; the landlord is not responsive to requests for repairs, her new bike is stolen, and skirmishes over the towing of cars in the neighborhood (including Rayna’s) lead to her citation for misdemeanor disorderly conduct.

Along with gang violence and unresponsive landlords, Rayna also fights to protect her family from child welfare services (DCF). Years ago, they took one of her sons for 3 months when she missed three doctor appointments. She fired her public defender and successfully represented herself in getting him back. When another son was injured by a classmate, Rayna called DCF to investigate the school, but they also investigated her. Not trusting the schools to protect her children, she has been homeschooling them ever since.

Ultimately, Rayna dreams to escape the gaze of the social welfare system altogether and become a homeowner. She explains that she has learned from her past efforts to protect herself and her children from abusive partners, gangs, and neighborhood violence, that landlords find it easier to blame and get rid of the tenants than to support them in pressing charges against abusers. Even more important, she feels that Section 8 keeps her under a watchful gaze: “They [Section 8] can look at anything that has to do with Rayna…I don’t want to have to give them my Social, my kids birth certificate, my income. You have to give them all your life—your guts, your ribs—everything.”

While she struggles to make her life in New Haven, Rayna also longs for companionship. At baseline, she has just broken up with Randall, a man 10 years her junior who had spent much of their 2 years together in prison. During the study, Rayna and Randall got back together and broke up again multiple times. She said he was smart and interesting, enjoyed doing things with her, and she could talk with him about anything. But he had trouble finding work, and when he did it was low paid, short-lived, or inconvenient (e.g. early hours). And, he continued to “smoke weed and hang out with his boys.” Most damaging were two acts that broke her trust. First, she discovered that “he was sleeping with a girl around the corner… I knew he had a friend around the corner, but..he didn’t make it seem like it was a *relationship*. But I begin to realize in our relationship he was creating arguments and … leaving easy with no problem…” So she investigated and confirmed her suspicions. Even more than a sexual betrayal, she felt that it signified his unwillingness to work on their relationship: “I’d ruffle him up and he goes over there where it’s easy and then… when he wants me to talk… he calls me.” In addition, he slashed her tires in retaliation for breaking up with him: “…after you stab my tires, there’s no more trust there. I would never put money in a bank account with you, you might get upset and take it all.” Their pattern of breaking up and getting back together persisted until Christmas of 2019. They had not spoken in the 5 months since.

Rayna expressed her sense of the potential for their relationship in her first interview, “I had such big plans for [Randall]… He used to be so smart, but he changed.” She blamed the change on his 17-month incarceration, which, she said, turned him into an “alpha male…Something happened” that made him need to prove himself with women. Whether prison had this effect or not, his involvement with the CL system clearly shaped the possibilities for their relationship, in spite of their efforts. His record made it difficult to get the good jobs that Rayna wanted for him, and, she suspected, sent him to his existing networks in the illegal economy to make money. Given his recent criminal record, he likely did not qualify for housing assistance, or believed he did not [44]. But Rayna had no intention of jeopardizing her family’s housing by putting him on her lease, or even letting him live with her: “He has to live somewhere else.” So, with limited access to independent and affordable housing of his own, Randall moved among the homes of women who did have such access, further undermining the progress of Rayna’s relationship work with him.

In reflecting on their relationship in her last interview, Rayna captures the potential it held for her, as well as its ultimate ending. “[M]ore than ever I miss his smile. Like it was just like a warm, happy feeling, that we were happy to be in each other’s presence.” Still, she conceded, “he’s not as strong as I am, he’s like one of my sons, someone I have to encourage and uplift… I’m looking at him like, ‘you didn’t come along. You were supposed to jump along with me…you were supposed to be [by] my side, not in front of me, not behind me.”

She spoke with far less regret when looking back on her relationship with Chilton, who she met soon after confronting Randall over the tire-slashing. Chilton too had a criminal record. Initially she describes him as a “sweetheart” and someone who she enjoys sex with. But she also complains that he has “no personality, no talk” and acts strangely when he stays at her house, hoarding his own food and obsessing when she moves his things. She once angrily walked him out of her home: “…the time that I allow you to have, you appreciate it. … I allow you in my space but when you do that in my space, bye.” When he’s not staying with her, Chilton is at his mother’s house, or, she suspects but doesn’t really care, with other women. Though she considers the possibility of a long-term relationship with Chilton, his lack of personality and her unease with him in her home soon convinces her otherwise. But Rayna continues to allow him to spend nights. In the last interview, she provides further insight. “He was the most meek, shallowest person I ever met in my life,” but he provided some financial assistance. “I would tell him, you know, I’m about to go to sleep. … And so he would know that he couldn’t come [over to my place]. So, in order for him to try to, you know, manipulate me, so I wouldn’t have to spend my money on groceries or whatever …he would give me money …that’s the only reason why I made it, but once I started working the crazy hours and saving my own money I stopped [letting him come over].”

Rayna is aware that having sex with multiple men can put women at risk for HIV—her aunt and uncle died from AIDS. And she indicates using condoms early in her relationships with both men. But over time, condom use interfered with her quest for a “forever partner.” When angry with Randall, she withheld sex altogether rather than insist on condoms. When she discovered his relationship with the neighborhood woman she said, “Ewww!…. I would rather masturbate or have no sex [than have sex with him].” Randall would try other approaches to retrieve her favor: “‘Still [no sex]?’ ‘Nope.’ [H]e was like, ‘Well, maybe money will help,’ and I was like, ‘Money doesn’t do it for me either.’ [Laughs] ‘Money comes and goes. It’s not like a good memory we had.’” Eventually she forgives him and they return to their past sexual practices. Similarly, as she sought to determine whether Chilton could be a long-term partner, she let condoms go; once it became clear he couldn’t, the precedent had been established for them both. Furthermore, to uphold her own self-image, Rayna draws clear boundaries around who she will sleep with. When Chilton once pressed her on whether she was sleeping with other men she snapped, “What? If I let myself go and let everybody I met talk to me and-and pump on me I wouldn’t’ even be a good person, would I?.. I wouldn’t be worth having sex with today now would I?” In this way, Rayna distinguishes herself from those who she thinks may need to worry about their sexual health.

## Relationship Work, Social Context, and HIV

These three narratives illustrate how women’s relationship work is shaped in a context characterized by housing vulnerabilities, the many manifestations of mass incarceration and the ever-present surveillance state. They also suggest how the meanings of sexual practices are constructed in this context, within the work of relationship building and maintaining. In so doing, they direct attention away from sexual risk as contained in individual behaviors and towards understanding social context as “risk.”

Lily’s case study demonstrates some of the challenges of maintaining a long term committed partnership during a household separation—a separation she perceived as having resulted from her husband’s betrayal of their relationship. As she works on her relationship, Lily simultaneously prioritizes the care of their children. Lily and her husband’s relative success in navigating the social context is likely built on their respective access to affordable housing. Additionally, they have resources to support separate households. Her husband has food stamp benefits for the children, and a steady job, perhaps reflecting his limited interaction with the CL system, that provides enough income to pay for his and the children’s housing and contribute towards financial support of his wife. Lily has a housing voucher that she maintained while in prison awaiting sentencing, in part due to the understanding of her landlord. These circumstances are relatively rare in the context in which Lily and her husband live. And, theirs is a fragile security. To introduce condoms into this situation, given the meaning both she and her husband give them, is to threaten both the trust built through their relationship work and, potentially, their children’s well-being (which Lily believes would be impacted were the marriage to end). Indeed, as Lily notes, if either of them mentioned condoms it would signal a change in the meaning of that relationship—suggesting another perceived betrayal that might be impossible to overcome.

Like Lily, Maya worked to preserve her committed relationship. According to her definition of commitment, that work simultaneously involves caring for her husband’s health. Relative to Lily, she and her husband have far fewer resources to facilitate this work. Whereas the fracture of Lily’s household resulted from a perceived act of betrayal, for Maya, it was her and her husband’s commitment to each other that changed their living arrangements. Yet her relationship work, along with searching for housing and employment, seems undermined at every turn. Likely recognizing the fragility of their situation, they have established rules about how to navigate sex with other partners while leaving their commitment to each other intact. In spite of their foresight and efforts, their relationship ends. We don’t know what happens to Maya’s husband. But Maya embarks on a new relationship with a past love, perhaps building on a pre-existing foundation of earlier relationship work. Still, the contextual strains placed on this new relationship are markedly similar to those that led to the dissolution of her previous one: a partner with severe health issues, a recovery threatened by the pervasive presence of drugs, and CL stipulations that, in requiring regular negative drug tests lest she be sent back to prison for 7 years, threaten to undermine their commitment to each other. While Maya’s overlapping relationships and non-condom use may increase her HIV-related risk, they also signify her dogged pursuit of a committed partnership meant to protect both her and her husbands’ health and well-being.

Rayna worked to establish a committed relationship with Randall, and for a time, to determine if Chilton had potential to be such a partner. She has made a life for herself and her children that revolves around maintaining a safe and stable household and avoiding the gaze of the CL and social service systems. Keenly aware and fiercely protective of this fragile stability, she restricts both men’s access to her home. They can stay but they “need somewhere else to live.” But where do they live in a context where their access to affordable housing is shaped by their incarceration history? Furthermore, that access is gendered: women are more likely than men to have subsidized affordable housing, in part, because they are caretakers of children and less likely than men to have been incarcerated [45]. Unlike Maya, Rayna is not interested in taking care of her partner; she wants someone who is her equal, and a trustworthy companion and provider. Yet in this social context, it is hard for Randall to meet her standards. Condoms are a part of the work of establishing and developing her relationships. At the start, like Lily and Maya, she and her partners use them, but continuing to insist on their use over time is both a sign that the relationship may not be progressing and a barrier to its further progression. Though generally aware of the risks associated with unprotected sex with multiple partners, Rayna’s self-image and history with these two men mean for her, that she is not having “risky sex” with multiple partners. Still, she indicates that she gets tested as part of her annual doctor visits. Testing negative confirms her sense that she is not at risk. Ultimately, neither of Rayna’s relationships progresses. At her last interview, she has succeeded in keeping her family together and maintaining their well-being. But this tenuous success is tempered by the lack of a partner’s companionship in her life.

## Discussion and Implications

Repeated, longitudinal qualitative interviews provide understanding of the complex ways that HIV related sexual practices are (a) embedded in relationships that are themselves embedded in broader structures of social inequality and (b) produced by the dynamic intersection of these broader structures with the work of building, maintaining, and ending relationships. Our focus here is on heterosexual relationships, as understood and described from the perspective of the women in them, and as interpreted within our own analytical framework. We do not claim to necessarily understand participants’ relationships or sexual practices, or the processes that have shaped them, in the same way participants would understand them. We do attempt to be clear about our framing and to provide compelling evidence in support of our interpretations.

Our analysis contrasts with social ecological conceptualizations of “the social determinants of health,” which typically view HIV (and other health outcomes) as the product of health-related risk behaviors that are independent of but determined by “upstream” social factors (e.g. [7–9]). They rarely consciously theorize the social “determinants” on which they focus; instead defining them as “social” because they are not individual behaviors. And, in separating risk behaviors from their “social” determinants they neglect the ways they can derive meaning from and give meaning to the “social.” Thus, such analyses cannot fully capture the nature and range of interventions needed to address and prevent HIV.

We apply a “social determination” framing [12, 13] to understand women’s HIV “risk” as it is embedded within and produced by mass incarceration and housing vulnerabilities, two contemporary manifestations of historically rooted systems of race, class, and gender inequality in the United States. In so doing, we have also contributed in several ways to a growing literature on mass incarceration and HIV. First, when operationalizing mass incarceration, this literature has focused almost exclusively on self or partner incarceration, demonstrating associations with or the pathways through which they produce “HIV risk”: unprotected sex, higher numbers of lifetime partners, concurrency, relationship instability, or partnership dissolution [34–38]. We have instead conceptualized mass incarceration broadly, as it informs life in poor, segregated, urban neighborhoods and in turn, shapes vulnerability to HIV among the women who live there. In the words of Michelle Alexander, writing in the foreword to Schenwar and Law’s book, *Prison By Any Other Name* [46]: “…’mass incarceration’ should be understood to encompass all versions of racial and social control wherever they can be found, including prisons, jails, schools, forced ‘treatment’ centers, and immigrant detention centers, as well as homes and neighborhoods converted to digital prisons.”

Second, we have added to the understanding of how mass incarceration shapes life in these neighborhoods, and HIV in particular, by drawing attention to its intersection with housing vulnerabilities, which similarly represent a long history of policies and practices that reflect race, class and gender interests and reproduce new forms of race, class and gender inequality [17, 45]. Limited access to safe and affordable housing is but one signifier of these vulnerabilities. In no state, is full-time minimum wage work sufficient to rent an unsubsidized fair market two-bedroom unit [47]. Waitlists in New Haven average 10,000 households long with only 400 cycling out annually [48] and city residents face challenges accessing subsidies [49] that are exacerbated for those with a CL history [44].

Among the themes that emerge in the narratives of Lily, Maya, and Rayna that provide further insight into the complex ways their relationship work is shaped by the intersections of mass incarceration and housing vulnerabilities, three are particularly noteworthy: state surveillance, including but not limited to carceral control; tenuous and conditional access to stable housing; and caretaking of self and others. To preserve her long-term relationship with her husband and protect herself from carceral control, Lily removed him from the lease and limited her contact with him, including sex, during her probation period. At the same time, she continued caring for their children on weekends and over the summer. These actions may have protected her committed relationship, but they also posed potential threats to it, by providing him opportunities to find other partners. Her access to housing helped make their arrangement possible; but that access was not unconditional. Though her housing voucher was transferrable, changing landlords would subject her to criminal background checks, but staying with her current landlord, meant fewer housing options. So far, he has been unable to offer her a larger home in a neighborhood suitable for the whole family. In the meantime, Lily relies on the length of time they have been together to confirm that their relationship remains a committed and monogamous one.

Maya and her husband left their subsidized housing as a way to continue to live together. But finding a new place that would make this possible was made challenging by criminal background checks and a new eviction record. Without a subsidy, the need for her to find a job was all the more important. But caring for her disabled husband restricted her job and housing searches and ultimately appears to have been a final factor in the dissolution of the relationship. For Maya, his return to drug use signified her own failure to protect him. Still, throughout their bouts of homelessness, household separation, and time together in a house that strained their budget and a neighborhood that jeopardized their efforts to stay off drugs, Maya relies on their agreed upon rules for sex with others to protect their committed “monogamous” relationship. At its ending, her work to (re) establish a committed relationship with a previous partner is interrupted when she resumes substance use, violating the conditions of her probation. To avoid 7 years of prison, Maya agrees to enter a treatment program. But, to continue her relationship work with the new partner, she selects a program located in a neighborhood associated with her history of substance use, rather than one in a different town. She fears that being too far away from him will jeopardize her relationship work even as she recognizes that the closer program jeopardizes her recovery success.

Though Rayna herself did not have a CL history, over 20 years of protecting herself and her children from the violence of the streets and former partners has brought her to the attention of the police. Run-ins with the social welfare system, as well as her self-advocacy and engagement of legal aid in resisting them, has made her all the more leery of state surveillance, even as it also likely brings her further under its gaze. And Rayna’s partners certainly had histories of CL involvement that interfered with her relationship work by, for example restricting their access to job opportunities and housing. Along with protecting them (and herself) from state surveillance, Rayna’s commitment to her children’s welfare required that she maintain access to subsidized housing. But it also interfered with her relationship work. Rayna expressed a clear desire for a committed monogamous relationship with a “forever partner”—an equal, who she could wake up to in the morning and go to sleep with at night. And though Randall seemed to have this potential she could not risk establishing a household with him, where they could sleep and wake up together, until she was certain. In other circumstances, they might have lived separately as they worked to build their relationship, but Randall’s housing options were limited; he stayed (and had sex) with other women who, like Rayna, also had access to housing. They continued to try to move their own relationship forward. But at the same time, given Randall’s relationships with others, she engaged in another sexual relationship (with Chilton) as part of her effort to find a forever partner. Condom-free sex was critical to Rayna’s work in pursuit of a long term committed partnership, as was her own sense that she carefully selected potential long-term partners. She confirmed her belief in the safety of this approach with annual HIV tests.

The lives of Lily, Maya, and Rayna are fundamentally shaped by the intersecting impacts of mass incarceration and housing vulnerabilities, which, among other things mean they are subjected to various forms of state surveillance, provided only conditional access to safe and affordable housing, and incompletely supported in their multiple caretaking responsibilities. Their narratives demonstrate how life in this context shapes and perpetuates race, class, and gender inequities. They also suggest that what may appear like concurrency, relationship instability, multiple partners, and unprotected sex—“HIV risks”—may be better understood as their work to establish and maintain monogamous committed relationships (the types of relationships that are “safest” from the standpoint of HIV). Under the conditions in which they live, their relationship work both advances and undermines these goals.

## Conclusions

Our effort to detail these women’s relationship work and related challenges suggests both that their relationship work *is* the work of HIV prevention, and how life in low-income, segregated New Haven neighborhoods *is* their HIV risk. This has at least two critical implications for addressing HIV related sexual risk. First, interventions to change individual behaviors must recognize how the meaning of those behaviors is constructed within relationships that are, in turn, embedded in specific social contexts. Prevention interventions are also part of this context and have implications for the meaning given to relationships.

Second, however well-informed of social meanings and context such individually focused interventions are, they are not likely to eliminate race, gender, and class inequities in HIV. This goal will require a focus on the structures that underly those inequities and ultimately, the creation of new contexts that ensure health for all. We have focused on two contemporary manifestations of more deeply rooted systems of inequality: mass incarceration and housing vulnerabilities. They operate via policies, programs, practices, and decisions, that can be changed. For example, federal policies restrict access to housing based on CL history; landlords and employers use background checks to determine access to housing and jobs; zoning policies contribute to neighborhood segregation; child welfare services prioritize investigation of mothers while accepting the word of school administrators; community resources are spent on promoting “safety” through the surveilling of low-income neighborhoods, patrolling of schools, and incarcerating of low level drug crimes. But efforts to change these and other relevant policies must also consciously acknowledge and challenge how they represent more deeply rooted systems that reflect and protect race, class and gender interests and power. The work of reducing or eliminating HIV transmission generally and inequities in transmission in particular is inextricably linked to the work of confronting these systems.
