# Correction to: Social Determination of HIV: Women’s Relationship Work in the Context of Mass Incarceration and Housing Vulnerability

**DOI:** 10.1007/s10461-021-03299-5

**Published:** 2021-05-15

**Authors:** Kim M. Blankenship, Alana Rosenberg, Danya E. Keene, Akiv J. Dawson, Allison K. Groves, Penelope Schlesinger

**Affiliations:** 1 Department of Sociology, American University, 4400 Massachusetts Ave NW, Washington, DC 20016-4072 USA; 2 Department of Epidemiology of Microbial Diseases, Yale School of Public Health, Yale University, New Haven, CT USA; 3 Department of Social and Behavioral Sciences, Yale School of Public Health, Yale University, New Haven, CT USA; 4 Department of Sociology, Howard University, Washington, DC USA; 5 Department of Criminal Justice and Criminology, Georgia Southern University, Statesboro, GA USA; 6 Department of Community Health and Prevention, Dornsife School of Public Health, Drexel University, Philadelphia, PA USA

## Correction to: AIDS and Behavior 10.1007/s10461-021-03238-4

The article “Social Determination of HIV: Women’s Relationship Work in the Context of Mass Incarceration and Housing Vulnerability”, written by Kim M. Blankenship, Alana Rosenberg, Danya E. Keene, Akiv J. Dawson, Allison K. Groves, Penelope Schlesinger was originally published electronically on the publisher’s internet portal on 1st April 2021 without open access. With the author(s)’ decision to opt for Open Choice the copyright of the article changed on 30th April 2021 to © The Author(s) 2021 and the article is forthwith distributed under a Creative Commons Attribution.

The original article has been corrected.

